# Barriers and Enabling Factors for Taking PrEP: The Lived Perspectives of Female, Male and Transgender Sex Workers in Thailand

**DOI:** 10.1007/s10461-026-05124-3

**Published:** 2026-05-12

**Authors:** Ravipa Vannakit, Surang Janyam, Chamrong Phaengnongyang, Chomnad Manopaiboon, Inthira Suya, Terence Beney, Philippe Girault, Stephen Mills, Michael Cassell, R. Cameron Wolf, Jintanat Ananworanich

**Affiliations:** 1 Bangkok Interdisciplinary Research and Development, Bangkok, Thailand; 2 Amsterdam Public Health, Amsterdam University Medical Center, University of Amsterdam, Amsterdam, The Netherlands; 3 Amsterdam Institute for Global Health and Development, Amsterdam, The Netherlands; 4 Amsterdam Institute for Immunology and Infectious Diseases, Amsterdam, The Netherlands; 5 Service Workers In Group (SWING) Foundation, Bangkok, Thailand; 6 Independent Consultant, Paris, France; 7 FHI 360 Asia Pacific Regional Office, Bangkok, Thailand; 8 FHI 360 Headquaters, Durham, NC, 27701 USA; 9 AIDS Action Baltimore, Baltimore, MD USA

**Keywords:** PrEP, Female, Male and transgender sex workers, HIV prevention, Thailand, LAI-PrEP, Stigma and discrimination

## Abstract

Despite Thailand’s goal of ending AIDS by 2030, pre-exposure prophylaxis (PrEP) uptake remains very low among sex workers (SWs) as they are effectively excluded from programming. This qualitative study explores PrEP pereceptions, decision-making and long-acting injectable (LAI)-PrEP potential among 113 female, male and transgender SWs across seven high-burden provinces. Utilizing 13 focus group discussions and thematic analysis guided by the Health Belief Model, findings revealed uneven and limited PrEP awareness, with many female SWs encountering PrEP for the first time. Key barriers identified by SWs included HIV-related stigma, lack of appropriate information and issues regarding eligibility. Among transgender women sex workers, there are concerns about hormone therapy interactions. Conversely, high perceived risk and family responsibilies motivated interest in taking PrEP. Participants strongly preferred LAI-PrEP for its discretion and compatibility with irregular sex work schedules. Enhancing equitable access requires decentralized, community-led delivery that addresses structural vulnerabilities including criminalization, stigma and discrimination. While LAI-PrEP addresses adherence challenges, its success depends on intensified awareness and targeted information provision, affordability and integration with existing community-led or key population-led health services to ensure culturally appropriate, stigma-free and sustainable HIV prevention for sex workers.

## Introduction

Pre-exposure prophylaxis (PrEP) is an evidence-based biomedical intervention that can reduce the risk of HIV transmission by up to 99% when taken consistently [1–3]. The World Health Organization (WHO) recognizes PrEP as a core component of combination HIV prevention, strongly recommending its use for individuals at substantial risk of HIV infection, including sex workers (SWs), as part of a public health approach [4]. The introduction of long-acting injectable (LAI) PrEP, a new and effective HIV prevention technology, presents a promising opportunity to rapidly reduce new infections and end AIDS by 2030 [5].

Sex work significantly shapes Thailand's economy and public health landscape. The sex worker population, comprising female sex workers (FSWs), male sex workers (MSWs) and transgender women sex workers (TGSWs), is disproportionately affected by HIV due to structural, social and behavioural risk factors [6–8]. Despite its significant economic contributions, sex work remains criminalized, which exacerbates adverse health outcomes by reinforcing SWs’ reluctance to seek care and assert their rights when they are violated. SWs face elevated risk of HIV exposure due to inconsistent condom use, high numbers of sexual partners and socio-economic vulnerabilities such as income precarity, housing insecurity and reliance on clients for economic survival, all of which can constrain their ability to negotiate safe sex or to access needed health care services.

Oral PrEP was formally introduced and recommended in Thailand's National Guidelines for HIV Prevention in late 2014/early 2015, following discussions and trials starting around 2010. Pilot programmes were launched in 2015 and later scaled up, integrating it into national HIV services and eventually universal health coverage (UHC). As of early 2024, there were a total of 32,661 active users, with men who have sex with men (MSM) the primary user group with an estimated 85–90% of all users, while transgender women (TGW) account for 10–15% of all users [9]. This means that PrEP uptake among SWs, especially FSWs, but also among MSWs and TGSWs, is historically low (< 1%, 4.5% and 2–3% respectively). Among FSWs, the estimated range of users is from < 500 to 1,000 [9, 10].

Consistent with global trends, PrEP uptake among SWs remains limited due to persistent structural and social barriers [9]. While oral PrEP is available free of charge, SWs face a range of interlocking barriers to PrEP uptake, including criminalization, stigma, discrimination, gender-based violence and limited access to healthcare services [11, 12]. A significant barrier to expanding uptake is the fact that SWs are not included among the 6 priority populations for PrEP programming in the National PrEP Guidelines (2021) [13]. These are: (i) discordant couples, (ii) MSM; (iii) TGW; (iv) adolescents; (v) pregnant women; and (vi) people who inject drugs. Gaps persist in reaching these specified target populations with tailored PrEP interventions. Lack of provider training in PrEP delivery and insufficient integration of PrEP into community-based services continue to impede equitable access [14–16].

The introduction of new PrEP options including LAI-PrEP presents a promising opportunity to address some of these barriers [17]. Injectable PrEP offers advantages such as reduced dosing frequency and increased discretion, which may appeal to SWs with irregular schedules or concerns about pill visibility [2, 18–21]. However, successful implementation requires addressing systemic issues such as affordability, accessibility and health education to increase demand, countering misinformation and misconceptions [22–24]. Including SWs among the target populations for national PrEP programming would be an important step forward.

This study explores the low uptake of PrEP among SWs in Thailand, investigating facilitators and barriers in programming, PrEP decision-making and the potential of injectable PrEP in more effective service delivery. The approach taken is grounded in the principle that consulting marginalised groups such as SWs is essential for devising policies and programmes that are both effective and respectful of human rights. For effective, truly empowering and protective human rights-based policies to be developed and implemented, it is indispensable that greater attention and visibility be given to their voices and rights. To do so, SWs in all their diversities and their representative organisations must be duly consulted and included in the policy-making process, as their experiences and perspectives are essential for developing effective policies and interventions that support their human rights and dignity [25, 26]. SWs have unique first-hand knowledge of the environment in which they work, the risks they face and their health and safety needs. Inclusion is a fundamental requirement of a human rights-based approach ensuring that policies and programming do no harm.

We aimed to elucidate how SWs perceive the needs, risks, benefits and social dynamics that will likely shape PrEP uptake [27–29]. The findings aim to enhance PrEP policy and programming to support accelerated uptake among all sex worker sub-populations.

## Methods

### Research Design and Study Setting

In 2023, a study titled “Investigating Factors Influencing Decision-Making on Pre-Exposure Prophylaxis (PrEP) Use Among SWs in Thailand” was conducted involving a cross- sectional survey administered to 1,511 SWs across seven high HIV-burden provinces representing all regions of the country. These were: Bangkok, Chonburi, Chiang Mai, Kanchanaburi, Phuket, Sa Kaeo and Udon Thani. In addition to the survey, this study was conducted to delve deeper into SWs’ awareness, knowledge and willingness to use PrEP in the selected provinces.

We chose a qualitative design consisting of 13 FGDs, of which 5 groups comprised FSWs, 4 groups of MSWs and 4 groups of TGSWs. Each FGD consisted of 5–13 individuals and were conducted from September to December 2023. We selected the Health Belief Model (HBM) as a qualitative research tool as a framework to support the documentation and analysis of sex worker perspectives on PrEP. The HBM provides a structured framework to incorporate the specific perceptions and unique barriers of marginalized populations, such as SWs in Thailand [24]. By identifying these beliefs, we can develop targeted, culturally appropriate interventions that support the meaningful participation of these communities in PrEP programming [30].

The HBM helped to reveal the perspectives of sex worker groups on PrEP. The focus on “perceived barriers” (e.g., cost, stigma, or general mistrust in the healthcare system due to past discrimination) allowed us to pinpoint practical challenges faced by sex worker populations. Understanding these specific barriers is crucial for developing effective interventions and studies have shown perceived barriers to be the most powerful predictor of preventative health behaviours across various populations [27]. The model was also used to explore and understand the specific socio-cultural attitudes and beliefs related to perceived susceptibility, severity and benefits within the different marginalized sex worker subgroups. This moves beyond generic commonplace assumptions and allows for a nuanced understanding of specific sex worker health beliefs. The HBM-guided research incorporated qualitative methods to gather rich, lived experiences directly from our respondents.

While the HBM identifies individual perceptions, critics note that it may under-represent broader systemic factors such as poverty and lack of resources [31]. Therefore, addressing PrEP uptake in Thailand requires not only changing individual beliefs (quick wins) but also implementing structural 'system shifts' that address the socio-economic barriers faced by SWs.

### Sampling Technique

Participant recruitment was organized by SWING. Interested sex worker volunteers from various locations, including entertainment venues, massage parlours, streets and community-based organization outreach sites were invited to participate in FGDs. The participants included both venue-based, non venue-based SWs and online SWs. In each province, 1–2 FGDs were conducted according to the estimated size of the number of SWs in the area.

### Sample

113 participants were drawn from a larger cross-sectional survey on PrEP use among the three sub-groups of SWs aged 18 years or older who provided informed consent. Among the participants, FSWs represented the largest group (43 individuals: 38.1%), closely followed by MSWs (39 individuals: 34.5%) and TGSWs (31 individuals: 27.4%). See Table 1.

**Table 1:** Sex worker participants by sub-population

Group	Province	Sub-population	Number of participants	Age range	Date
1	Chonburi (Pattaya)	FSW	9	28–58	23/11/2023
2	Kanchanaburi	FSW	11	26–59	26/9/2023
3	Sa Kaeo	FSW	10	20–39	6/10/2023
4	Udon Thani	FSW	7	19–53	7/11/2023
5	Bangkok	FSW	6	37–52	16/11/2023
6	Chonburi (Pattaya)	MSW	10	22–50	24/11/2023
7	Bangkok	MSW	6	21–49	17/11/2023
8	Phuket	MSW	13	23–46	21/11/2023
9	Chiang Mai	MSW	10	20–48	13/12/2023
10	Chonburi (Pattaya)	TGSW	13	23–58	23/11/2023
11	Bangkok	TGSW	8	25–34	17/11/2023
12	Phuket	TGSW	5	23–51	21/11/2023
13	Chiang Mai	TGSW	5	19–45	13/12/2023

### Data Collection

To ensure both confidentiality and comfort, FGDs were held in private settings, such as designated meeting rooms or offices, providing a secure environment for discussion. Each session lasted between 1.5 to 2 h and was audio-recorded with participants’ consent.

The conversations were moderated by a trained facilitator using a semi-structured questionnaire framework designed to encourage in-depth discussions on several critical topics. Participants shared their awareness and understanding of PrEP, including newer forms such as injectable PrEP, discussed the benefits and barriers to its use, explored the triggers that might prompt uptake and offered valuable suggestions on how to improve access and uptake of this important preventive measure.

The semi-structured guide was designed to mitigate confirmation bias by starting with general inquiries into the socio-economic lived experiences of SWs following the COVID-19 pandemic. Questions moved from broad health-seeking behaviors to specific PrEP perceptions only after establishing a community-driven context. This approach ensured that structural vulnerabilities—such as the need to negotiate fresh sex for higher pay or the logistical hurdles of mobile work—emerged naturally from the participants’ narratives rather than being forced into HBM categories.

### Data Analysis

Audio recordings were transcribed verbatim in Thai and subsequently translated into English; accuracy was verified through review by bilingual members of the research team. Data were analyzed using thematic analysis, employing a hybrid inductive and deductive approach. An initial codebook was developed deductively based on the six constructs of the HBM. Two independent coders (including a community-based peer researcher from SWING) established the coding framework. New codes were added inductively to capture emergent structural themes such as transphobia in healthcare and lifestyle fit. Themes were validated using a group-based pile-sorting technique. Grounded in the methodology of Weller and Romney (1988) [32], this allowed participants to prioritize barriers such as “pill burden” versus “provider attitude”. Data recorded and notes from the focus groups were examined and information gathered during the discussions was consolidated and summarized for all sex worker groups participating in the sessions. Key terms were coded and concepts were identified based on the recurring themes that emerged from the discussions.

The analysis was guided by the six key constructs that influence an individual’s decision to act in the HBM [33, 34]. Specifically, the model helped examine participants’ perceived susceptibility to HIV, the severity of HIV, perceived benefits of and barriers to PrEP use, external cues to action (e.g., peer influence, campaigns, online messaging) and self-efficacy in PrEP decision-making.

## Ethical Considerations

The study received ethical approval from the Institutional Review Board (IRB) of the Institute for the Development of Human Research Protections (IHRP) on September 4, 2023 (Protocol No. IHRP2023114). All participants were fully informed about the study’s purpose and procedures, provided their consent in accordance with ethical standards and were compensated for their time (500 baht). Additionally, consent for audio recording was explicitly requested and granted before each session. The FGDs were managed with sensitivity to the risks of participant recognition as a SW and confidentiality was assured throughout the process. Post FGD referral pathways to services that may be needed were shared with participants. The findings of the study were shared with SW communities through SWING.

## Limitations

Several limitations should be considered when interpreting the findings of this study. First, recruitment through SWING introduced a sampling bias toward SWs already connected to peer support; thus, the perspectives of non-networked, undocumented, or rural workers may be underrepresented, affecting broader transferability. Second, preferences for LAI-PrEP remain largely hypothetical. As many participants (particularly FSWs) were first introduced to PrEP during this study, stated interest reflects anticipated acceptability rather than observed clinical uptake or long-term adherence. Third, the use of FGDs in a stigmatized context may have introduced social desirability bias, potentially influencing disclosures regarding sensitive behaviors. Logistical challenges, such as workplace-related late arrivals and environmental noise, also impacted data collection sessions. Finally, while the HBM provides a structured framework, its individualistic focus may overlook broader systemic factors. We mitigated this by integrating structural issues such as criminalization and socio-economic precarity as prompts in the FGD sessions.

## Results

The transcription of the FGDs were analysed in line with the 6 components of the HBM framework.

### Perceived Susceptibility to HIV

Perceived susceptibility to HIV is a highly important factor for people considering taking PrEP, significantly influencing their willingness to initiate and adhere to the medication. Perception of risk emerged as significant in PrEP decision-making, regardless of SW sub-population. This was influenced by perceived risks arising from experience of sex work. These included potential exposure to HIV infection and sexually transmitted infections, a large number of clients, intoxication, unprotected sex activities, condom breakages, limited trust in sexual partners, accepting clients known to have HIV and gender-based violence (GBV).

*“Being service workers (i.e. SWs) makes us more inclined to use PrEP because we feel like we’re taking risks. We have very clear risks, and we should also use condoms”* (TGSW, Bangkok).

The recognition of risk and vulnerability had motivated some participants to consider or actively take PrEP as a harm-reduction strategy.

*“I decided to take it because I live in this kind of society”* (MSW from Pattaya), referring to the gay community where he frequently has many opportunities for sexual encounters, whether selling sexual services or not.

*“We have a chance to take risks, so I think it's better to prevent (HIV) than to fix”* (MSW, Pattaya).

Some participants did not perceive themselves to be at significant risk of HIV, which influenced their decisions not to take PrEP. This was linked to personal preventive behaviours or a weaker identification with sex work as a primary occupation. Some preferred to opt for condoms for protection.

*“Some people don’t take PrEP because they don’t think they’re at risk. I feel that I always take good care of myself so there is no need for me to take PrEP”* (FSW, Pattaya).

### Perceived Severity

Perceived severity of HIV is a highly important factor for people taking or considering PrEP, as it significantly influences their motivation, willingness to initiate PrEP and adherence to the regimen [35].

The participants’ perception of the potential consequences of contracting HIV may influence their decision to pursue PrEP. For instance, concerns about the impact on the wellbeing of their family members if they did not take care of themselves, can play a pivotal role in their decision-making.

*“I am interested in taking PrEP. I am worried that in the future, if I have children, they will be infected with HIV. I am planning to take PrEP every day. Taking PrEP is confirmation that we are not HIV positive. It sets the standard”* (FSW, Udon Thani).

*“I have a five-year-old child, so I will do everything to protect myself. I worry about my young child”* (FSW, Bangkok).

The consideration of potentially losing their sex work career due to HIV can influence their PrEP decision-making.

*“If it's AIDS, it's game over. We don't even know what kind of infection we might get. If we run into something serious like AIDS, we can't keep offering our services and making money”* (TGSW, Chiang Mai).

### Perceived Benefits

Perceived benefits are highly important for people taking PrEP, often serving as the primary motivation for use. While the core benefit is HIV prevention, users are strongly driven by associated social and emotional (collateral) benefits, which can outweigh potential barriers like stigma or side effects [36].

Multiple participants, MSWs, FSWs and TGSWs, cited the occupational risks inherent in sex work as a driving motivation for taking PrEP. For MSW participants, PrEP offers added protection alongside condoms, reinforcing their sense of security from infection. This in turn can enhance sexual enjoyment by making it worry-free. Many acknowledged PrEP as a critical safeguard, especially in cases of condom breakage, non-use, client deception or when clients offered more money for condomless sex. The primary benefit is that PrEP provides effective protection against HIV transmission.

*“The primary advantage of PrEP is interrupting HIV transmission, preventing further spread. If this medication had been available earlier, transgender individuals might not have experienced such high infection rates”* (TGSW, Chiang Mai).

Respondents were more favourably inclined towards PrEP use when they understood its benefits and perceived it as essential for safeguarding their health and livelihood. A clear recognition of PrEP efficacy in preventing HIV emerged as a pivotal factor influencing SWs’ decisions to initiate or continue its use.

*“Initially, I decided to take it because I heard it prevents HIV. While I do use condoms, it's not foolproof”* (MSW, Chiang Mai).

FSWs discussed how PrEP use connected directly with their responsibilities as family caregivers and income providers, underscoring their need to stay healthy. Good health, free from HIV, was also seen as essential for a long working life.

*“I must be cautious and protect myself. I don’t want my family to suffer”* (FSW, Bangkok).

There was strong interest expressed in LAI-PrEP, should it become available and affordable. Participants highlighted this option as a means to overcome multiple challenges associated with daily oral PrEP, particularly those related to adherence, stigma and practicality. LAI-PrEP, administered monthly or bi-monthly, was viewed as a more sustainable alternative. Ensuring affordability and providing accurate, culturally attuned education will be essential for successful implementation. The reasons for the preference for LAI-PrEP were primarily centered around convenience. Many participants described difficulty maintaining a consistent medication routine due to unpredictable or long work schedules.

*“Yes, if this is available (injectable PrEP), we will consider taking it. It sounds convenient.”*

*(FSW, Bangkok).*

Discretion was another key factor. Several participants raised concerns about the visibility of oral PrEP, which could invite stigma or uncomfortable questions. LAI-PrEP, compared by some to contraceptive injections, was seen as less conspicuous. Additionally, LAI-PrEP was seen as a way to reduce the burden of managing multiple daily medications, particularly for TGSWs already adhering to hormone therapy. For SWs who travel regularly, LAI-PrEP was also viewed as more practical and less likely to trigger scrutiny during cross-border checks. While enthusiasm was widespread, participants expressed varied opinions on pricing. Notably, some participants associated injectable formulations with greater potency or efficacy, a perception that aligns with common beliefs that injections are more effective than oral medications. At the same time, others emphasized the need for clear communication and education about the duration of protection and clinical effectiveness of LAI-PrEP.

### Perceived Barriers

Perceived barriers to PrEP services are a significant issue because they directly contribute to low PrEP uptake and adherence, which in turn hinders effective HIV prevention efforts and perpetuates health disparities. These barriers prevent individuals who could benefit most from PrEP from accessing and using the medication consistently [37].

Awareness of PrEP was found to be limited among all SW sub-groups. FSW participants consistently reported lower levels of awareness than MSW and TGSW. For nearly all participating FSWs, the FGD was their first introduction to PrEP. They have little access to information about PrEP and consequently have very limited PrEP awareness and knowledge in different settings**.**

*“We don’t know about this prevention method. We don’t have the information”* (FSW, Bangkok).

*“If PrEP has been available for ten years, we definitely think it is good. But I had never heard of it”* (FSW, Udon Thani).

In Chiang Mai, one of the first cities in Thailand to promote PrEP among LGBTQI + , some TGSW participants had a very limited knowledge of PrEP. This appears to indicate only partial coverage of this key population.

MSW participants showed higher levels of awareness and were relatively well-informed. In Pattaya, they were knowledgeable about PrEP and included current users. They cited various MSM community information sources such as dating apps (e.g., Blue Hornet) and gay health segments on television.

Despite years of PrEP availability in Thailand, misunderstandings about its purpose remain widespread among SWs. A common misconception was that PrEP is a treatment for HIV-positive individuals, rather than a preventive measure.

*“I thought you had to be infected to take PrEP”* (TGSW, Chiang Mai).

Some participants viewed PrEP simply as “medicine”, which they associated with illness. This led to reluctance to take it when feeling healthy. This framing undermines understandings of PrEP as a preventive tool.

*“No matter how you say it, it is still a medicine. I don’t want to take medicine”* (MSW, Pattaya).

Some participants also reported that others in their community assumed anyone taking PrEP must already be HIV-positive, reinforcing misinformation and discouraging uptake.

Stigma emerged as a powerful deterrent to PrEP uptake. Many feared negative judgement from peers, clients, family, or healthcare providers. Among MSW, using PrEP was sometimes perceived as signaling promiscuity or an intent to engage in unprotected sex.

*“People will think we are infected”* (MSW, Pattaya).

*“I’m afraid people will see me as a sexual maniac”* (MSW, Pattaya).

*“Those who take PrEP look like they are ready for sex without condoms”* (MSW, Pattaya).

Others expressed fears that being seen with PrEP would result in being mistaken for someone living with HIV. For others, popular knowledge of use of PrEP might lead to being recognized and outed as a SW. TGSW participants were particularly concerned about losing clients if found to be using PrEP. Healthcare-related stigma was also reported. Some feared judgmental attitudes from medical staff or encountering acquaintances at hospitals when picking up PrEP medication.

*“I don’t want people around me and my family to know I receive PrEP because I don’t want them to know I am a sex worker”* (FSW, Sa Kaeo).

*“Maybe customers who don’t really know about PrEP will think we’re infected and disappear. Even if this perception is corrected later, they won’t come back”* (TGSW, Chiang Mai).

Participants identified several practical challenges related to oral PrEP uptake. Many found it difficult to maintain a consistent daily routine due to irregular work hours and unpredictable schedules. TGSWs voiced concern about adding PrEP to existing medication routines, especially those involving hormone therapy.

*“I’m not consistent with taking PrEP on time, especially with customers”* (MSW, Chiang Mai).

“*I take a lot of medicines already, and I’m worried about side effects if I add PrEP”* (TGSW, Pattaya).

Accessing PrEP posed logistical difficulties. Long waits, limited service hours and centralized hospital-based delivery discouraged some from starting or continuing use. Participants called for expanded access through local clinics or mobile services. Compared to condoms, oral PrEP was perceived as less convenient. Its bulkier packaging, need for daily adherence and greater visibility have made it harder to integrate into mobile lifestyles.

*“PrEP comes in a bottle—it’s not as easy to carry as condoms”* (MSW, Chiang Mai).

Widespread concerns about potential side effects emerged as a notable barrier to PrEP uptake. Participants across all SW groups expressed fears about how the medication might affect their health, often based on second-hand information or community rumours. For some, the idea of adding PrEP to existing medication routines, particularly for TGSW taking hormone therapy, felt overwhelming.

*“I think there are definitely side effects, even birth control pills have them”* (TGSW, Chiang Mai).

*“I have never taken it because I was afraid of the side effects”* (TGSW, Chiang Mai).

Many worried about long-term damage to the liver and kidneys, while others raised concerns about taking PrEP with alcohol. Participants voiced anxieties about changes in appearance. Reports of hair loss, weight loss and darker skin tone circulated among peers. A few mentioned more speculative fears, like fatigue, reduced sexual function or drug interactions. While some with direct experience of PrEP reported few issues, most participants remained cautious, often lacking access to reliable medical information to address their concerns.

*“I’m curious about its impact on the liver and kidneys”* (MSW, Chiang Mai).

*“I heard it might cause your skin to get darker”* (FSW, Pattaya).

*“I take a lot of medicines already*, *and I’m worried about side effects if I add PrEP”* (TGSW, Pattaya).

### Cues to Action

Cues to Action (CTAs) are extremely important for PrEP, acting as triggers like a partner's support, clinic reminders, or seeing ads that prompt people to start or stay on PrEP, but their effectiveness hinges on accessibility and relevance; consistent supply, supportive partners, clear health messaging and reduced barriers (cost, location) are crucial for these cues to overcome barriers such as stigma or confusion, turning awareness into consistent action [38].

The lack of basic knowledge about PrEP services was recognised as a major constraint to improving uptake among SWs. This is particularly acutely felt among FSWs. It was argued that improved information provision is needed to address prevailing misconceptions about PrEP and to respond to the potential stigmatization of users. Practical information is also needed about the benefits of PrEP and how to access it.

TGSWs and MSWs, in particular, expressed a strong preference for community-led clinics over hospitals. They felt more comfortable in these settings due to increased confidentiality and reduced fear of stigma.

*“Society still considers us strange. So I don’t want to go to the hospital”* (MSW, Phuket).

*“Ladyboys have easier access to the Rainbow Sky Community Health Centre* (a community-based clinic). *We rarely go to the hospital because they will think that we have AIDS even though we don't have it yet”* (TGSW, Phuket).

The discussions revealed demand for services beyond hospitals, including district health hospitals, mobile services and proposed radical solutions such as PrEP vending machines or dispensing availability at convenience stores. This highlights the need for greater accessibility and convenience, especially for those who face challenges with time and travel. In addition, concerns were raised about judgmental attitudes from some healthcare providers, lengthy wait times and complicated processes in hospitals. Participants suggested separating blood tests from medication pick-up and reducing the number of questions to make the experience more efficient and less intimidating.

*“Some people wanted to take it, but the hospital makes it complicated. Doctors don't easily prescribe the medication. There are many processes and they ask a lot of questions, like “Why do you want to take it?”* (FSW, Pattaya).

Peer influence plays a critical role in shaping attitudes toward PrEP among SWs. Across all SW groups, participants consistently emphasized that knowing someone who used PrEP increased their own openness to it.

*“I don’t hesitate. A friend recommended it, and since my friend takes it, I feel at ease and take it too”* (TGSW, Pattaya).

*“If your friends take it, it will be easier to decide to take it”* (FSW, Udon Thani).

Among MSWs, peer norms were especially influential. This underscores the powerful impact of social networks in normalizing PrEP and reducing hesitation. For many, a recommendation from a peer carried more weight than formal health messaging. Visible peer use helps normalize PrEP and lowers perceived barriers.

*“If all your friends take PrEP, we would feel like a black sheep”* (MSW, Pattaya).

There was interest expressed in the development and implementation of active campaigning to promote PrEP among all SWs to raise awareness of delivery modalities and the range of benefits for users as well as correcting misconceptions. Some participants felt that the campaign should extend more generally to all individuals who perceive themselves to be at risk.

### Self-efficacy

Self-efficacy is an individual's confidence in their ability to access, initiate and consistently take PrEP as prescribed [39]. It is a primary driver of PrEP uptake and adherence among key populations. It manifests through various stages of decision-making, from the initial choice to start treatment to the ongoing management of occupational risks [40]. Self-efficacy is linked to developing essential behavioral skills, such as effectively communicating about sexual health with partners or providers, which are crucial for successful PrEP use and overall sexual health [41]. Higher self-efficacy helps individuals maintain a positive self-image and focus on the health benefits of PrEP, rather than being deterred by negative social perceptions [42].

*“I don’t want to take PrEP. I’m not fully committed to sex work yet”* (FSW, Kanchanaburi).

Self-efficacy was revealed in individual statements about decision-making connected with initiating PrEP and managing their health. Personal responsibility for family members was particularly cited by FSWs as a key factor in decision-making.

*“I decided to take it because I heard it prevents HIV”* (MSW, Chiang Mai).

*“I take PrEP for prevention, and I don't care about others' opinions. I am the primary breadwinner for my family, so I must be cautious and protect myself. I don't want my family to suffer”* (FSW, Bangkok).

*“If you’re not taking care of yourself, no one else will”* (FSW, Bangkok).

A strong sense of personal responsibility and self-efficacy emerged among MSWs, FSWs and TGSWs in managing their health, particularly in relation to HIV prevention. Participants acknowledged their occupational risk and consistently emphasized the importance of taking proactive measures, such as regular testing and consideration of PrEP. In addition to personal agency, participants highlighted the influence of peer networks in shaping attitudes toward PrEP, suggesting that self-efficacy is not only individually internalized but also socially reinforced within supportive community contexts.

*“Doing this kind of work means looking out for yourself. If we slip up, there’s no going back.*

*We’ve got to love and protect ourselves” (MSW, Chiang Mai).*

## Discussion

Our findings demonstrate the importance of obtaining and documenting the lived experiences of marginalized populations, such as SWs, as a fundamental requirement for ensuring equitable access to health services. Despite the proven efficacy of PrEP and its free of cost availability under UHC in Thailand, lived narratives can help reveal the implementation gap that conditions real-world uptake through the intersection of factors such as criminalization, stigma and economic precarity. Documenting these intersections enables more holistic service delivery development.

By centering sex worker voices, this study moves beyond asking why communities are hard to reach and instead interrogates how health systems must adapt to be more accessible. In this study, the HBM serves as a powerful diagnostic lens, helping the transition from identifying *what* the barriers to PrEP uptake are to understanding *how* they function, informed by the lived realities of different sex worker subgroups. This structured approach ensures that the voice of marginalized SWs is not just heard, but translated into actionable data that highlights exactly where the disconnect between policy intention and community reality exists. By systematically deconstructing participant narratives into constructs such as perceived barriers and cues to action, the framework reveals why clinically effective tools like PrEP may be perceived as socially or practically inaccessible and what needs to be done to address this. The HBM was instrumental in uncovering how the perceived benefit of HIV protection is frequently weighed against the “perceived barrier” of stigma or medical interference—such as the specific concern among TGSWs regarding hormone therapy interactions.

This research provides a first comparative analysis of FSW, MSW and TGSW perspectives in Thailand in relation to PrEP service delivery (See Table 2). The comparative HBM analysis reveals that while the perceived severity of HIV is high across all subgroups, it is viewed as an existential threat to their roles as family breadwinners. The path to PrEP as HIV prevention is obstructed by distinct, population-specific barriers. For FSWs, the primary obstacle is an “information desert” arising from historical PrEP programming focused on MSM, whereas for TGSWs, the barrier is physiological, centered on the need for hormonal harmony between PrEP and transition-related care. Amidst these challenges, LAI-PrEP emerged as a widely favoured intervention across all sex worker groups due to its convenience, discretion and ability to reduce daily adherence burdens, especially for those managing hormone regimens or unpredictable/long hours of sex work. Across all groups, the transition to self-efficacy is mediated by the service delivery environment. A sharp contrast emerged between unwelcoming hospital environments and friendly community-led models. Participants identified hospital-based services as a major deterrent due to long wait times, complicated registration processes and perceived judgmental attitudes from medical staff. Furthermore, the fear of being outed as a sex worker in a public hospital setting remains a powerful barrier. Conversely, CBOs provide a stigma-free, confidential environment where peer-led outreach fosters trust and simplifies the service delivery processes. These findings suggest that equitable PrEP scaling in Thailand requires moving beyond a one-size-fits-all approach to embrace decentralized, subgroup-specific strategies that address these unique cognitive and structural realities.

**Table 2:** PrEP Perceptions among Thai sex worker sub-population

HBM Construct	Female SWs (FSWs)	Male SWs (MSWs)	Transgender SWs (TGSWs)
Perceived susceptibility	Very low Awareness: Often first-time exposure to PrEP during FGDs; “Information desert”	Higher Awareness: Information accessed via specialized apps and LGBTQ + networks	Higher Awareness: Digital connectivity to TGW health networks
Perceived severity	High: Fear of losing breadwinner status and ability to support family	High: Concern regarding health impact on long-term earning capacity	High: Fear of losing breadwinner status and economic independence
Perceived barriers	Lack of outreach; geographic barriers; institutional stigma in hospitals	Client pressure for “fresh sex”; fear of being "outed" in clinical settings	Hormonal Harmony: Fear of drug interactions with gender-affirming care
Perceived benefits	Occupational Insurance: Protection against client-driven risks	Discretion; freedom from daily pill burden (if using LAI-PrEP)	Integrated care; protection that doesn't compromise transition goals
Self-efficacy	Low due to lack of information and access	Moderate; depends on negotiation power with clients	Moderate; enhanced by peer support but hindered by “pill burden”
Cues to action	Mobile clinics; peer-led community outreach	LGBTQ + dating apps; word-of-mouth in venues	Integration with hormone therapy; CBO-led “safe spaces”

## Conclusion

Thailand stands at a pivotal juncture in its HIV response. Biomedical advances, particularly LAI-PrEP, offer significant potential to close the gap in HIV prevention; success, however, depends on dismantling the structural and informational barriers that continue to exclude SWs. This study demonstrates that low PrEP uptake is not merely a result of individual beliefs but is a consequence of systemic failures, including the historical exclusion of SWs from national programming and the persistence of medicalized, often stigmatized, service delivery models.

Our findings advocate for a structural, intersectional and community-driven approach to PrEP implementation. Based on the perspectives of SWs, PrEP modalities must adapt to the diverse lifestyles of sex worker subgroups. For TGSWs, integrating PrEP with gender-affirming care (e.g., hormone therapy) reduces clinical burden and enhances uptake. For FSWs, expanding mobile and peer-led outreach is essential to bridge gaps in awareness and geographic access. CBOs should be the primary frontline providers. Peer-led social media campaigns can further normalize PrEP as a standard tool for occupational health. SW PrEP programmes need to be integrated with interventions addressing income precarity and GBV. These social determinants directly shape risk perception and the capacity for sustained engagement with prevention tools.

To ensure the sustainability of these improvements, SWs need to be formally recognized as a distinct, prioritized target population within the National PrEP Guidelines to ensure dedicated resource allocation. The focus of PrEP delivery should be shifted from centralized hospitals to community-led clinics and pharmacies. This decentralization is critical to reducing the institutional stigma that currently deters many SWs from seeking care. Fast-tracking the introduction of LAI-PrEP as a discreet, high-adherence option should be considered. Implementation must prioritize community-led distribution for SWs to prevent the emergence of new health disparities.

Ultimately, the question is no longer why SWs are not using PrEP, but what are the steps the public health system must take to make diversified targeted programmes that can reach all sex worker sub-populations. Achieving the goal of ending AIDS by 2030 requires a transformation in the core values of PrEP service delivery, ensuring more equitable access to PrEP services for all sex worker sub-populations.
